# From Petri Plates to Petri Nets, a revolution in yeast biology

**DOI:** 10.1093/femsyr/foac008

**Published:** 2022-02-10

**Authors:** Stephen G Oliver

**Affiliations:** Department of Biochemistry, University of Cambridge, Sanger Building, 80 Tennis Court Road, Cambridge CB2 1GA, United Kingdom

In the beginning was… the genome.

The 30th of January, 1991 found me outside Temple Underground Station on the Thames Embankment in London. John Sgouros of the Martinsried Institute for Protein Sciences, who had flown over from Munich that morning, emerged from the station carrying seven A0 sheets in a roll. They were copies of Figure 1 for the paper ‘The complete DNA sequence of yeast chromosome III’, for which we were determined to get a 1991 submission date. We walked around the corner to the *Nature* offices in Little Essex Street and handed in these giant Figures together with envelopes containing the manuscript and the other Figures, which I had brought down from Manchester. *Nature* then lost the copies of Figure 1 but, by 27th March 1992, the paper was accepted and appeared in the journal on the 7th of May 1992 (Oliver *et al*. 1992). This paper reported the first complete DNA sequence of a chromosome from any organism and, with it, everything changed—certainly for me, for yeast genetics, but also for the wider biomedical research community's view of the value of genome sequencing. The complete sequence of this chromosome taught us some important lessons in two major areas.

First, in the area of eukaryotic chromosome organisation and evolution. We found that the relationship between the genetic distance between loci, as measured in terms of recombination frequency, and that of physical distance, as measured by the DNA sequence, was far from linear. In fact, the ratio of the genetic distance (in cM) to the physical distance (in kb) showed a > 40-fold range for different intervals along the chromosome, being smallest close to the centromere and greatest half-way down each chromosome arm (Figure 3 in Oliver *et al*. 1992, 1993). It also indicated the role of retrotransposons in the generation of redundancy in the yeast genome by duplicating portions of the chromosome (Wicksteed *et al*. 1994) and increasing the copy number of tRNA genes (Eigel and Feldmann 1982, Genbauffe *et al*. 1984). Redundancy is the substrate exploited by evolution to generate novel functions (Ohno 1970, and see below).

Second, the complete sequence of this chromosome taught us that we knew much less about the genetics of *Saccharomyces cerevisiae* than we thought. The yeast genetics research community, at this time, was justly proud of the genome map (Mortimer *et al*. 1989) that had been constructed by classical genetic techniques supplemented by recombinant DNA analyses. In fact, there was even talk that for some functions, e.g. DNA repair, the map was saturated. However, inspection of the chromosome III sequence showed that there were 182 potential protein-encoding genes (open reading frames, ORFs, > 100 amino acids in length) of which only 37 were known from classical studies (Oliver *et al*. 1992). There were 35 laboratories involved in the sequencing of this first chromosome, and the Yeast Genome Consortium, ably led by André Goffeau, went on in this ‘cottage industry’ approach to sequence the other 15 chromosomes until the first complete genome sequence of a eukaryotic organism was published in 1996 (Goffeau *et al*. 1996), just a year after that of the first complete bacterial genome sequence (Fleischmann *et al*. 1995).

There were individual papers reporting the sequence of the other 15 chromosomes (Dujon *et al*. 1994, Feldmann *et al*. 1994, Johnston *et al*. 1994, Bussey *et al*. 1995, Murakami *et al*. 1995, Galibert *et al*. 1996, Bowman *et al*. 1997, Churcher *et al*. 1997, Dietrich *et al*. 1997, Jacq *et al*. 1997, Philippsen *et al*. 1997, Tettelin *et al*. 1997), many of these were published, together with a bioinformatics overview (Mewes *et al*. 1997), in a special issue of *Nature* entitled *The Yeast Genome Directory* (for which the European Commission paid handsomely) that did not appear until the following year. The regular issue of *Nature* that appeared at the same time carried a ‘News & Views’ piece on the yeast genome from Craig Venter and his colleagues (Clayton *et al*. 1997) that, in the short run, got more attention than any of those in the *Directory*. I think that it is difficult for today's researchers, used to the facility of next-generation sequencing and automated methods of genome assembly, to appreciate just how much work was put in by the different yeast labs involved in the project. When the project began, the sequencing was done by hand (only 7% of the chromosome III sequence was obtained using automated methods (Oliver *et al*. 1992) and sequence assembly was largely a hand-crafted exercise. Although all yeast researchers, and very many others, make direct or indirect use of the yeast genome sequence every day, it has received the ultimate accolade of ‘citation eclipse’, being rarely referred to in their papers.

## Functional genomics

The complete yeast genome sequence revealed 6200 putative genes encoding 5885 proteins, 275 tRNAs, and 40 small nuclear (sn) RNAs (Goffeau *et al*. 1996). This may be compared to the 769 loci on the *S. cerevisiae* genetic map at the outset of the sequencing project (Mortimer *et al*. 1989). Bioinformatic analyses performed at the time of completion of the genome sequence (Mewes *et al*. 1997) revealed that only *ca*. 40% of the genes revealed by the sequence could be attributed a function, either from direct experimental evidence, or their products’ membership of well-defined protein families, or their significant amino-acid sequence homology to proteins of known function.

Thus, 7–8 years of genome sequencing had not only revealed eight times as many genes than 40 years of classical analyses, but a large proportion of these putative genes appeared to be completely unknown to biological science.

Even with just the sequence of chromosome III in hand (some 30 years ago), it was immediately obvious that the normal course of genetics would need to be turned on its head. Instead of isolating mutants with defective or altered phenotypes and using classical genetic analyses to define functional genes and map their relative locations on chromosomes, now genes would be both identified and mapped by genome sequencing, and it would be necessary to work in the opposite direction to discover their functions (Oliver *et al*. 1993, Oliver 1996a, 1997). It was also evident that this new approach to genetic analysis would require techniques that were every bit as comprehensive as that of genome sequencing itself. By 2000, it was possible to lay out the basic levels of ‘omic analysis of genome, transcriptome, proteome, and metabolome (Oliver 2000).

The magnitude and complexity of the task of elucidating the functions of all the novel genes revealed by the complete yeast genome sequence suggested that such an enterprise was beyond the skills and resources of any single laboratory or institution and that we should build on the network approach to large-scale projects that had been the vision of André Goffeau. Accordingly, a new European Consortium, EUROFAN (European Functional Analysis Network; https://cordis.europa.eu/project/id/BIO4950080), comprising some 145 laboratories from 14 countries, was established at the beginning of 1996 with the aim of determining the role of 1000 *S. cerevisiae* genes of unknown function that had been identified from the genome sequence (Oliver 1996b). The basic strategy was to generate a library of single-gene deletants with which to seek for null phenotypes. Following the generation of the mutants, in which all laboratories participated initially, EUROFAN did phenotypic analyses that were organised in a hierarchical manner that was designed to approach the biological function of each gene with ever-increasing specificity as it moved down the pathway of analysis.

The deletion mutants were generated by PCR-mediated gene replacement using the *kan*MX cassette constructed by Achim Wach and Peter Philippsen (Wach *et al*. 1994). This cassette is designed such that it has no sequence homology anywhere in the yeast genome. This means that it can be targeted to a specific chromosomal location by PCR-generated extension sequences that are homologous to sequences flanking the ORF, i.e. to be deleted. Cells with this cassette successfully integrated could be selected using the drug geneticin (G418), resistance to which was conferred by the *kan*^R^ gene carried by the cassette. This cassette, itself, conferred no measurable phenotype on the recipient strain other than drug resistance (Baganz *et al*. 1997). The phenotypic analyses undertaken were both qualitative and quantitative in nature. While competition experiments were identified as being particularly useful for quantitative analyses, the initial methods of discriminating between mutant strains in mixed cultures were rather cumbersome (Baganz *et al*. 1998). This problem was quickly resolved by Ron Davis's innovation of incorporating gene-specific molecular bar-codes into the PCR primers used to amplify the deletion cassettes (Shoemaker *et al*. 1996). This meant that each deletant was uniquely identified by a pair of bar-codes and the advantages of this were so obvious that a new international project was formed between labs in the US, Canada, and EUROFAN that resulted in the generation of a complete library of strains containing bar-coded deletions for all protein-encoding genes predicted from the genome sequence (Giaever *et al*. 2002).

At the outset of these phenotypic analyses, the most obvious phenotype was cell death, and *ca*. 19% of yeast's protein-encoding genes were found to be essential, *i.e*. haploid segregants lacking the gene fail to form a colony on a YEPD agar plate. However, it should be recalled that essentiality, like any other phenotype, is context dependent. The ORF YDL120 provides a good example of the way this hierarchical analysis of gene function worked, and a rare case where a given gene was taken all the way through the hierarchy of tests and the detailed biochemical lesion resulting from the deletion was not only identified, but also related to a human disease. Initial tests on a ydl120Δ mutant showed that it failed to grow on glycerol, and so it was passed to the Mitochondrial Node for more detailed analysis. There, Francoise Foury and her colleagues were able to locate the fault to mitochondrial iron transport. Moreover, the amino-acid sequence of this yeast ORF's protein product showed similarity to that of the human protein frataxin, which is the product of the human gene determining the neurodegenerative disease, Friedreich's ataxia. The yeast lesion could be complemented by the expression of a cDNA copy of the human coding sequence, confirming functional homology and elucidating, for the first time, the biochemical basis of the human disease (Foury and Cazzalini 1997, Rotig *et al*. 1997).

Quantitative phenotypic analyses exploited the unique identifiers that the molecular bar-codes provided in order to carry out competition experiments between the complete library of deletion mutants, usually as heterozygous (hemizygous) diploids so that all protein-encoding genes should be interrogated. Studies were carried out both in batch (Deutschbauer *et al*. 2005) and continuous (Delneri *et al*. 2008) culture. The latter (Pir *et al*. 2012) had the advantage of allowing competitions to be carried out under different nutrient limitations or at different growth rates (using chemostat culture) or in nutrient-unconstrained conditions (using turbidostat culture). Genes whose hemizygous mutants showed a significant change in their growth rate, compared to the wild type, were termed high flux control (HFC) genes. These HFC genes may be divided into two classes: a haploinsufficient (HI) set, where the hemizygous mutants grow slower than the wild type, and a haploproficient (HP) set, in which the hemizygotes grow faster than the wild type. The HI set is enriched for genes involved in the processes of gene expression, while the HP set is enriched for genes concerned with the cell cycle and genome integrity. Since haploproficiency was observed in turbidostat culture, this means that diploid cells lacking one copy of an HP gene grow at a rate in excess of the maximum specific growth rate previously observed in wild-type cells (Pir *et al*. 2012). This implies that the control of growth rate in yeast represents a trade-off between the selective advantages of rapid growth and the need to maintain the integrity of the genome.

For a subset of HP genes, heterozygous deletion was found to be sufficient to cause aberrant cell cycling and altered rates of apoptosis, phenotypes associated with cancer in mammalian cells (de Clare and Oliver 2013). Most of these yeast genes are the orthologs of mammalian cancer genes, and hence, our studies suggest that gene copy number variation (CNV) may lead to tumorigenesis in human cells. Using this yeast gene set as a model, it was shown that the response to a range of anticancer drugs is strongly dependent on gene dosage, such that low or intermediate concentrations of the drugs can actually increase yeast's growth rate. These data suggest that the identification of CNVs in tumor cells may assist both the selection of anticancer drugs and the dosages at which they should be administered. This is yet another example of how the systematic analysis of gene function in yeast (or functional genomics; Hieter and Boguski 1997) can shed light on human diseases.

For all its successes, EUROFAN's hierarchical approach to the elucidation of gene function did not decipher the role of as many of yeast's novel genes as had been hoped. This may be attributed to two main problems and yet more genome sequencing is providing at least a partial answer to them both. The first problem is our lack of knowledge of yeast ecology. This limits our ability to devise simple phenotypic tests at the lower end of the hierarchy of functional analysis. The second is the remarkable amount of redundancy in such a small eukaryotic genome as that of *S. cerevisiae*. Differences, between members of a gene family, in the regulation of gene expression or the cellular localisation of protein products can mean that this redundancy is more apparent than real (Delneri *et al*. 1999). However, a major source of redundancy in the *S. cerevisiae* genome is the whole-genome duplication (WGD) that the ancestor of this and related species underwent during their evolution. This major evolutionary event was first inferred by Ken Wolfe and Dennis Shields (Wolfe and Shields 1997 by analyzing the *S. cerevisiae* genome sequence (Goffeau *et al*. 1996) and was subsequently confirmed by sequencing the genomes of two members of the Saccharomycetaceae that diverged from the *Saccharomyces* lineage prior to the WGD—*Kluyveromyces* (syn. *Lachancea*) *waltii* (Kellis *et al*. 2004) and *Ashbya gossyppi* (Dietrich *et al*. 2004).

Since the discovery of the WGD, a huge amount of effort has gone into the isolation of strains of *S. cerevisiae* and its close relatives from the wild and the sequencing of their genomes. These efforts again involved a collaborative approach and the formation of research consortia such as Génolevures, led by Jean-Luc Souciet and Bernard Dujon (Sherman *et al*. 2004, Souciet *et al*. 2009), and the 1,000 Genomes project led by Gianni Liti and Joseph Schacherer (Peter *et al*. 2018). These studies have not only thrown light on the evolutionary history of the Saccharomycetaceae, including the domestication of *S. cerevisiae*, but the provenance of the wild isolates has also contributed to our understanding of yeast ecology. Despite all these efforts, both in the laboratory and the field, there remain some 18% of protein-encoding genes of *S. cerevisiae* that are still of unknown function (Wood *et al*. 2019). Such ‘orphan’ genes are often dismissed as being specialised genes that are unique to a particular species; in fact, more than one-third of them are conserved across the tree of evolution from yeasts to humans (Wood *et al*. 2019). Thus, the functional genomics agenda remains unfinished.

## Systems biology

The endpoint of the functional genomics agenda should be a complete list of the working parts of the yeast cell, such as you might find in the back of a workshop manual of a complex machine (Oliver 2002). However, this parts inventory is not sufficient to allow the servicing of the machine or to permit us to engineer it to fulfil new purposes. What is required is to know how those working parts interact to make a functional machine, and how those interactions are controlled; just as, for instance, we would need the wiring diagram of a radio in order to understand the interactions of its parts and how its performance is controlled (Lazebnik 2002). There were valiant attempts to take a systems approach to biology in the 1960s (see Waddington 1968). While it was recognised that this would involve the cooperation of experimental biologists and mathematical modellers, there were simply insufficient experimental data to permit the construction of robust and predictive models. In the event, the modellers went ahead without the essential data and, consequently, mathematical modelling got a bad name—at least among molecular geneticists.

One of the early steps required in a systems approach to yeast biology was to integrate the different levels of ‘omic analysis (genomic, transcriptomic, proteomic, and metabolomic) by carrying out what have come to be known as ‘multi-omic’ experiments (Castrillo *et al*. 2007). Since that time there have been enormous advances in the technology of ‘omic analyses, with the transition from microarrays to RNAseq for transcriptomic experiments (Waern *et al*. 2011, Hesketh 2019) and the extensive array of mass spectrometry techniques employed for both proteomics (Rees and Lilley 2011, Nightingale *et al*. 2019) and metabolomics (Winder and Dunn 2011, Chaleckis *et al*. 2019). For metabolomics, this has meant that the promise of being able to reveal gene functions by comparing the metabolic profiles of single-gene mutants (Raamsdonk *et al*. 2001) has at last been realised (Mülleder *et al*. 2016)—although it did require the conversion of the entire yeast deletion collection to prototrophy in order to achieve it (Mülleder *et al*. 2012).

For all the advances in these analytical technologies, it was two approaches that exploited the ‘awesome’ power of yeast genetics that made major contributions to the early development of yeast systems biology. The first of these was the yeast two-hybrid system developed by Stan Fields (Fields and Song 1989), which allowed the high-throughput analysis of protein–protein interactions. This could be applied, not only to yeast proteins (Uetz *et al*. 2000, Ito *et al*. 2001), but also to proteins of other species, *e.g*. humans (Rual *et al*. 2005), expressed in yeast. Whilst the system has its limitations, for instance it measures interactions within the yeast nucleus, a number of variations to circumvent them have been devised. For all that, Y2H analyses have provided a hugely valuable dataset when combined with studies that used other techniques (von Mering *et al*. 2002), including biochemical methods exploiting mass spectrometry (Gavin *et al*. 2002), and rigorous statistical analyses (Yu *et al*. 2008). The second approach has been arguably of even greater value since it identifies *functional* interactions. This is the high-throughput detection of gene-gene (epistatic) interactions by using the synthetic genetic array (SGA) methodology developed by Charlie Boone, Brenda Andrews, and colleagues in Toronto (Tong *et al*. 2001, 2004, Costanzo *et al*. 2010). At first, the synthetic phenotypes were monitored as a growth/no growth qualitative output, but subsequently colony growth rate was measured (Baryshnikova *et al*. 2010) and even cell morphology (Ohya *et al*. 2015, Mattiazzi Usaj *et al*. 2020). Similarly, the methods could, at first, only be applied to measuring interactions between pairs of nonessential genes but it has now been extended to include the essential genes through the use of titratable promoters (Mnaimneh *et al*. 2004) and temperature-sensitive alleles (Li *et al*. 2011). These methods of mapping both physical and functional interactions have proved enormously valuable to system modellers, not only to constrain their models, but also to test their predictions against empirical datasets (see below).

As with Functional Genomics, the advent of Systems Biology saw the establishment of networks of European yeast researchers to pursue major programmes of work—the Yeast Systems Biology Network (YSBN, led by Jens Nielsen; https://cordis.europa.eu/project/id/18942) and UNICELLSYS (led by Stefan Hohmann; https://cordis.europa.eu/project/id/201142). YSBN had a coordinating role in systems biology research using yeast as a model organism and aimed to employ both mathematical analyses and computational tools to integrate experimental data; it set out to develop common resources and standards. UNICELLSYS had the more focussed aim of achieving a quantitative understanding of how cell growth and proliferation are controlled and coordinated by both extracellular and internal signals. These two networks operated in a very synergistic manner.

Stefan Hohmann's organisation of UNICELLSYS showed considerable vision at a time when not everyone was convinced of the need for Systems Biology, or even that it represented ‘respectable’ science. Moreover, working together with Hiroaki Kitano, Stefan played a major role in coordinating and promoting Systems Biology on a global, scale. The work in UNICELLSYS involved some advances in proteomic technology to improve quantitation (Selevsek *et al*. 2015) and, also, a shift in emphasis from protein synthesis to post-translational modifications (Kapuy *et al*. 2009, Amoutzias *et al*. 2012) and protein–protein interactions (Nandy *et al*. 2010). Work on the responses to external stimuli concentrated on the response to osmotic stress, with experimentalists joining with modellers to elucidate the circuitry involved (Nadal-Ribelles *et al*. 2012, Tiger *et al*. 2012, Talemi *et al*. 2016). Work on the control of cell proliferation in response to internal signals concentrated on the cell cycle and had two strands—in one, the experimentalists worked in close collaboration with the modellers with some individuals becoming adept in both approaches (Barberis *et al*. 2011), while the other comprised a purely theoretical approach (He *et al*. 2011).

YSBN had the aim of establishing some common standards between research groups, not only in experimental methods (a problem already encountered in EUROFAN; Brown *et al*. 2001), but also in how both models and data were represented and stored. The standardisation of experimental methods (Canelas *et al*. 2010) was a massive undertaking that involved a comparison of the behaviour of two yeasts strains—CEN.PK113-7D, a lab strain that was widely used by yeast physiologists and that had already been extensively characterised by an earlier multi-lab experiment (van Dijken *et al*. 2000), and YSBN2, a new reference strain constructed in the s288c background (Winston *et al*. 1995) but with the auxotrophic markers replaced by wild-type copies of the genes. Batch and continuous fermentations were carried out in Jack Pronk's laboratory in Delft and samples distributed to the participating laboratories. These undertook analyses of the transcriptome (by four different methods in four laboratories), enzyme activities (three different protocols in two labs), and the metabolome (three different technologies in seven labs). This produced an extensive reference dataset that, regrettably has been underused (Canelas *et al*. has < 400 citations at the time of writing), perhaps because the transcriptome analyses were not carried out using RNAseq technology (Waern et al. 2011).

A major contribution of YSBN was to establish a consensus stoichiometric model of the yeast metabolic network (Herrgård *et al*. 2008). A genome-scale model of the yeast metabolic network had already been constructed by Jens Nielsen, Bernard Palsson, and their colleagues (Förster *et al*. 2003) building on Palsson's *E. coli* model (Edwards and Palsson 2000). However, the consensus model (which was constructed at a YSBN ‘jamboree’ in Manchester) benefitted from the input of a wide range of experts, standardised and unambiguous representations of chemical structures, its adherence to the Systems Biology Mark-up Language (SBML; Hucka *et al*. 2003), and its availability on a publicly accessible database (now at https://sysbiochalmers.github.io/yeast-GEM/). This model (Yeast1) was rapidly updated even during the course of YSBN (Nookaew *et al*. 2008, Dobson *et al*. 2010, Heavner *et al*. 2012), not least because it did not permit the performance of simulations and Flux Balance Analysis (FBA). The current version is Yeast8 (available at the website above) and the evolution of genome-scale models of the *S. cerevisiae* metabolic network is the subject of an excellent recent review (Yu *et al*. 2022).

Other systems of modelling yeast metabolism have been developed. Reiser *et al*. (2001) constructed a logical model of the yeast metabolic network. This is a very simple model: it has no dynamics and it does not even contain the stoichiometry of the reactions. However, each reaction is linked to a gene in the complete *S. cerevisiae* genome sequence (Goffeau *et al*. 1996) and this revealed a number of ‘orphan’ reactions, ones for which there was good evidence that they must occur in yeast, but for which no gene encoding the enzyme catalysing the reaction had yet been identified. Adam, a ‘Robot Scientist’, was then provided with this model and with access to the public sequence databases. This permitted Adam to use artificial intelligence to design, and robotics to execute, experiments that identified the genes associated with some of these orphan reactions—the first example of a machine discovering new science (King *et al*. 2009).

The most important developments of the genome-scale stoichiometric model of yeast metabolism have involved the exploitation of transcriptomic (Lee *et al*. 2012) or proteomic (Sánchez *et al*. 2017, Lu *et al*. 2019) data to further constrain the model and increase the accuracy of its predictions. Future developments are likely to involve the use of machine-learning techniques to reduce the uncertainties in enzyme kinetic constants, such as *k*_cat_ or *k*_m_ values (Kroll *et al*. 2021, Li *et al*. 2021). Uncertainties can be accommodated by other modelling systems such as Flexible Nets (a development from Petri Nets, hence the title of this piece), which can also deal with regulation of both reactions and the industrial processes which rely on them (Júlvez *et al*. 2018). In the other direction, Szappanos *et al*. (2011) used machine learning techniques to reconcile contradictions between the predictions of the yeast genome-scale metabolic model and the experimental genetic interaction data of Costanzo *et al*. (2010). In this way, they were able to correct errors in the model, demonstrating that there was only one route to NAD biosynthesis in yeast, and not two as there are in *E.coli* (Edwards and Palsson 2000). We can expect to see an increased use of machine learning and other forms of artificial intelligence in systems biology modelling. However, it is important that we ensure that our models have explanatory power as well as predictive accuracy.

## Final thoughts

Much of the work described in this article is the result of open-ended research programmes that aimed to produce useful data. In other words, they were hypothesis generating, rather than hypothesis testing. Science has always needed both these kinds of research (Kell and Oliver 2004), and data-generating research should not be dismissed as mere ‘stamp-collecting’. Indeed, early attempts to develop a systems approach to biology failed due to a lack of quantitative and comprehensive data (see above). Today, however, such data are in plentiful supply and there is much enthusiasm for investigations based on ‘Big Data’ (Pal *et al*. 2020). It is important to remember that the results of such analyses can be skewed by incomplete or biased data sets (for a topical example, see Bradley *et al*. (2021)). Moreover, despite the so-called ‘data avalanche’, there are data that are critical to the generation of accurate predictions by systems biology models that are either incomplete or unavailable. For example, our knowledge of the biochemical composition of yeast biomass under different physiological conditions is in a parlous state (Dikicioglu *et al*. 2015). Another crucial data set, the kinetic parameters of enzymes, will either be hard won through exhaustive (and exhausting) experimental work (Smallbone *et al*. 2013) or require the use of advanced deep-learning methods to leverage the scattered and heterogeneous experimental data available (Kroll *et al*. 2021, Li *et al*. 2021). Other essential resources that must be generated and maintained if the potential of Big Data analyses in systems biology is to be realised are properly curated databases—dumps of electronic lab notebooks are not useful. Such databases require not only the skills and commitment of professional curators, but also the direct involvement of experimentalists in the curation process (Oliver *et al*. 2016). A major problem is that the generation of these missing data and the maintenance of high-quality databases are often considered mundane activities that tend to be under-resourced by funding bodies. However, given the resources, the combination of the intrinsic advantages of yeasts as model organisms, and the demonstrated ability of the international community of yeast researchers to work together for the common good, will ensure that yeasts will remain in the vanguard of the revolution in biology and build upon the achievements of pioneers like Stefan Hohmann. Stefan was not only open to new technologies and new concepts in his science, but he was also open to collaboration and had a talent for organisation and coordination; we are all in his debt.
